# Expression and clinical significance of platelet-derived miR-145-5p and miR-6805-3p in diabetic kidney disease patients

**DOI:** 10.3389/fmed.2025.1529759

**Published:** 2026-01-12

**Authors:** Miao Hu, Yi Deng, Zhen Weng, Yujie Bai, Jiayan Zhang, Jianzhong Li, Lei Shen, Xiahong Shen, Ling Zhou

**Affiliations:** 1 Department of Nephrology, The First Affiliated Hospital of Soochow University, Suzhou, China; 2 Cyrus Tang Hematology Center, Soochow University, Suzhou, China

**Keywords:** diabetic kidney disease, platelet, miR-145-5p, miR-6805-3p, biomarker

## Abstract

**Objectives:**

Approximately 30–40% of diabetes mellitus (DM) patients will progress to diabetic kidney disease (DKD) and eventually cause end-stage renal disease (ESRD) during their disease course; therefore, early recognition and prevention of DKD is critical. The detection based on biomarkers derived from blood samples has the characteristics of easy availability and affordability. In addition to participating in thrombosis and hemostasis, platelets also have important secretion functions. Our research aims to identify novel platelet miRNA candidates for DKD diagnosis and to explore their clinical significance.

**Methods and results:**

miR-145-5p and miR-6805-3p were selected for further evaluation based on their differentially expressed status between DM and DKD subjects, as determined by both bioinformatic and RT-qPCR analyses, and their expression was significantly higher than that in healthy control and DM groups. Correlation analyses revealed that the levels of platelet-derived miR-145-5p and miR-6805-3p were positively associated with uric acid (UA), serum creatinine, blood urea nitrogen (BUN), urine albumin-to-creatinine ratio (UACR), cystatin C, platelet to lymphocyte ratio (PLR), and neutrophil to lymphocyte ratio (NLR), and negatively correlated with estimated glomerular filtration rate (eGFR), albumin, total cholesterol (TC), low-density lipoprotein cholesterol (LDL-C), blood glucose, and hemoglobin A1c (HbA1c). In addition, miR-145-5p expression level was positively correlated with globulin; the levels of miR-6805-3p but not miR- 145-5p were increased in DKD patients with higher staging.

**Conclusion:**

Our research suggests that platelet-derived miRNAs may be promising biomarkers for diagnosing DKD and monitoring its progression.

## 1 Introduction

Diabetes mellitus (DM) related microangiopathy that attacks the human kidney is the leading cause of chronic kidney disease (CKD) worldwide and will result in diabetic kidney disease (DKD) (1) and even end-stage kidney disease (ESKD) requiring renal dialysis or transplantation, thereby causing severe social and family burden (2). It is reported that approximately 30–40% of DM patients will eventually progress to DKD during their disease course (3). With the increasing incidence of DM worldwide, DKD-affected patients are also elevated (4). In China, significantly increased prevalence and incidence of DKD are observed, and it is estimated that the number of DM related DKD is about 24.3 million (5). Given the large number of patients, precise screening of high-risk patients with DM will be critical. Moreover, although proteinuria could be used effectively for monitoring DKD development and progression, its relatively late time window might hinder effective therapeutic intervention. In a hospital laboratory setting, blood-based testing, which requires minimal invasiveness, could be a better candidate for biomarker selection. In our previous studies, we obtained extracellular vesicles (EVs) from the plasma of DM and DKD patients and identified that EV-derived mRNA (AEBP1) (6) and miRNAs (miRNA-615-3p and miRNA-3147) (7) could differentiate DKD from DM and were involved in disease progression. However, EV isolation requires a commercial kit and a high-speed centrifugation device; it is necessary to discover novel biomarkers inexpensively. Genes (including mRNAs and miRNAs) and protein expression of blood-based biomarkers have been reported for DKD differential diagnosis; however, most of the current studies use the blood-derived nucleated cells, such as lymphocytes, monocytes, and neutrophils, for expression level evaluation. Less attention has been paid to the platelets, another part of blood composition, and a kind of anucleate cells. Compared with the time-consuming Ficoll reagent-based density gradient centrifugation and the expensive magnetic beads required for the isolation of blood-derived nucleated cells, platelet isolation is simple, high-yielding, and inexpensive, requiring only an inexpensive low-speed centrifugation device. Moreover, platelets have been implicated in several human diseases, including cancer (8), cardiovascular disease (9), and diabetes (10). Recently, increased evidence has shown that platelets play a role in the onset and progression of DKD (11). Multiple platelet-derived proteins are elevated in DKD and promote inflammation and fibrosis (12–15). Then the problem is to determine which genes or proteins to examine to obtain the diagnostic or high-risk differentiation information for DKD. With the development of high-throughput sequencing and protein mass technology, information on platelet-related genes and proteins can be easily obtained from multiple databases. Xie et al. (8) established a database called Platelet Expression Atlas (PEA) and described all the available platelet data from patients with different kinds of diseases, among which DM is included. In current clinical practice, renal biopsy, the gold standard for DKD diagnosis, is an invasive method for tissue. Although blood is a mixture composed of nucleated (white blood cells) and anucleate cells (red blood cells and platelets), platelet isolation can be achieved directly by simple centrifugation. Therefore, it is obvious that a platelet sample could be easily obtained (9). Moreover, previous studies (10, 11) indicate that platelets contributed to the development and progression of DM and renal disease. Building on our prior experience with miRNAs, we proposed evaluating the role of platelet-derived miRNAs in the diagnosis and treatment of DKD.

## 2 Materials and methods

### 2.1 Data acquisition and bioinformatic analysis

The project PRJEB18466 contains sequencing data for platelet-derived miRNA from three DM patients and three control subjects, which were downloaded from the European nucleotide archives (ENA; http://www.ebi.ac.uk/ena). The raw data were pre-processed by removing adapters using the Cutadapt software. Reads in the size range of 18–50 nucleotides were selected for mapping against mature miRNAs in the miRBase (version 21) using Bowtie, while the expression counts were obtained with Samtools (version 1.20). Differential expression analysis was performed using the DESeq2 package (version 1.38.3) on data from three DM patients vs. three control subjects or on two DM patients vs. two control subjects. Counts were normalized, and differential expression was inferred using the negative binomial distribution and a shrinkage estimator for the distribution variance of the counts.

### 2.2 Subjects

A total of 31 type 2 DM (T2DM) and 25 DKD patients were included from the Department of Endocrinology or Nephrology of the First Affiliated Hospital of Soochow University from October 2022 to March 2024 according to the 1999 WHO criteria for DM and the Chinese Clinical Guidelines for the Prevention and Treatment of Diabetic Kidney Disease (2019) for DKD. Moreover, patients were excluded when they met the criteria as follows: T1DM has a short duration (<10 years) or no diabetic retinopathy (DR); rapid estimated glomerular filtration rate (eGFR) decline: eGFR decline of ≥5 mL/min/1.73 m^2^ or eGFR decline of ≥25% from baseline within 1 year; rapid increase in urinary protein: (a) within a few weeks, 24-h-urinary protein increase by ≥50% from baseline, or by ≥500 mg/24 h in absolute terms; (b) at several weeks, urinary protein ratio creatinine (UPCR) increase by ≥50% from baseline, or by ≥500 mg/g in absolute terms (12); nephrotic syndrome; refractory hypertension; active urinary sedimentation; symptoms or signs of other systemic diseases; eGFR decline >30% within 2–3 months after ACEI or ARB treatment; abnormalities found by renal ultrasound; with coexisting other diseases, including hepatitis, tumors, autoimmune diseases, and hematological disorders. Moreover, 27 healthy controls were recruited from volunteers who underwent physical examination at our hospital. The 27 healthy volunteers had normal examination indices and had not been diagnosed with diabetes. The study protocol was approved by the Clinical Research Ethics Committee of the First Affiliated Hospital of Soochow University, and written informed consent was obtained from all the included subjects.

### 2.3 Clinical parameters

The clinical data for these patients included age, gender, total cholesterol (TC), triglyceride (TG), low-density lipoprotein cholesterol (LDL-C), high-density lipoprotein cholesterol (HDL-C), blood glucose, glycosylated hemoglobin (HbA1c), uric acid (UA), blood urea nitrogen (BUN), creatinine, urine albumin-to-creatinine ratio (UACR), 24-h urine protein (g/day), cystatin C, alkaline phosphatase (ALP), albumin, globulin, neutrophil/lymphocyte ratio (NLR), platelet/lymphocyte ratio (PLR) and monocyte/high-density lipoprotein ratio (MHR). The detailed information of these indices was shown in Table 1.

**Table 1:** Clinical indicators of the included subjects.

Clinical indicators	Health controls (*n* = 27)	DM (*n* = 31)	DKD (*n* = 25)	*p*-value
Gender (male, %)	14 (51.9%)	23 (74.2%)	19 (76.0%)	0.110
Gender (female, %)	13 (48.1%)	8 (25.8%)	6 (24.0%)	0.110
Age (years)	44.67 ± 11.26	48.35 ± 14.62	56.40 ± 10.52	0.004
TC (mmol/L)	4.98 ± 0.83	4.71 ± 1.78	4.32 ± 1.55	0.131
TG (mmol/L)	1.47 ± 0.61	1.70 ± 1.02	1.85 ± 1.18	0.379
LDL-C (mmol/L)	2.64 ± 0.62	3.18 ± 0.91	2.23 ± 1.12	0.001
HDL-C (mmol/L)	1.37 ± 0.26	0.94 ± 0.20	1.02 ± 0.42	<0.001
Blood glucose (mmol/L)	5.13 ± 0.85	7.93 ± 2.61	5.94 ± 1.69	<0.001
HbA1c (%)	5.20 (5.10–5.50)	10.30 (9.10–12.70)	6.60 (6.05–8.30)	<0.001
UA (μmol/L)	341.99 ± 80.43	343.20 ± 86.23	418.10 ± 86.48	0.002
BUN (mmol/L)	5.15 ± 0.82	5.56 ± 1.53	16.50 ± 7.73	<0.001
Creatinine (μmol/L)	64.20 (51.40–79.10)	58.10 (47.20–69.50)	242.70 (138.65–448.85)	<0.001
UACR	–	0.06 (0.04–0.13)	4.56 (2.70–6.06)	<0.001
24-h urine protein (g/day)	–	–	3.06 (1.90–4.59)	–
eGFR (CKD-EPI ml/min/1.73 m^2^)	105.80 (100.00–116.00)	108.10 (100.30–130.10)	22.20 (10.60–46.85)	<0.001
Cystatin C (mg/L)	–	0.91 ± 0.14	3.18 ± 1.73	<0.001
ALP (U/L)	73.96 ± 16.69	79.62 ± 26.52	74.30 ± 21.00	0.549
Albumin (g/L)	44.19 ± 2.02	40.48 ± 3.10	31.29 ± 6.11	<0.001
Globulin (g/L)	29.60 (27.80–30.90)	24.50 (22.00–27.10)	27.90 (24.05–30.30)	<0.001
NLR	1.48 ± 0.50	1.85 ± 0.85	2.97 ± 1.71	<0.001
PLR	107.20 ± 26.25	104.19 ± 44.38	139.52 ± 44.81	0.002
MHR	0.32 (0.22–0.35)	0.45 (0.40–0.58)	0.47 (0.38–0.64)	<0.001

### 2.4 Patient staging

Patients in the DKD group were divided into five stages based on eGFR levels. The specific classification basis is: G1 stage (>90 mL/min/1.73 m^2^); G2 stage (60–89 mL/min/1.73 m^2^); G3 stage (30–59 mL/min/1.73 m^2^); G4 stage (15–29 mL/min/1.73 m^2^); G5 stage (<15 mL/min/1.73 m^2^). Since there were only two G1 and G2 stage patients in the DKD group, we combined the G1 and G2 stages, representing the G1 + G2 stage (>60 mL/min/1.73 m^2^).

### 2.5 Blood collection and platelet isolation

A 5-mL citrate-anticoagulated venous blood sample was collected from the elbow veins of all the included subjects, and platelets were obtained according to a previous study. Briefly, Platelet-rich plasma was first obtained from 200 *g* centrifugation for 10 min, followed by a second centrifugation at 950 *g* for 10 min to obtain platelet pellets (13).

### 2.6 Quantification of platelet-derived miRNAs

Total RNA from the obtained platelet pellets was extracted using TRIzol reagent (Vazyme, Nanjing, China) according to the manufacturer’s instructions. Followed by RNA quantification using NanoDrop2000 (Thermo Fisher, CA, United States), miRNA 1st-Strand cDNA Synthesis Kit (Vazyme, Nanjing, China), and miRNA Universal (Synergetic Binding Reagent) SYBR for quantitative polymerase chain reaction (qPCR) Master Mix (Vazyme, Nanjing, China) were used for reverse transcription and real-time fluorescence quantification, respectively (real-time quantitative polymerase chain reaction [RT-qPCR]). The relative expression levels of miR-145-5p, miR-6805-3p, miR-144-5p, and miR-339-3p in platelets were calculated using the 2^−∆∆Ct^ method, while U6 was chosen as the internal control.

### 2.7 Statistical analysis

Statistics and graphs were performed using the Statistical Package for the Social Sciences (SPSS) version 25.0 software (SPSS, IBM, Armonk, NY, United States) or GraphPad Prism 9.1 (GraphPad, San Diego, CA, United States). Measurement data were expressed as mean ± standard deviation (SD) or median and interquartile range according to the distribution. Categorical data were expressed as numbers and percentages. One-way analysis of variance (ANOVA) and Kruskal-–Wallis rank–sum test were used for three or four groups of continuous variables comparison, and Tukey’s *post hoc* test was used for pairwise comparison. Pearson correlation and Spearman correlation analyses were used to assess the correlation. In addition, *p* < 0.05 was considered statistically significant.

## 3 Results

### 3.1 Platelet-derived miRNA candidates screening

**Figure 1:**
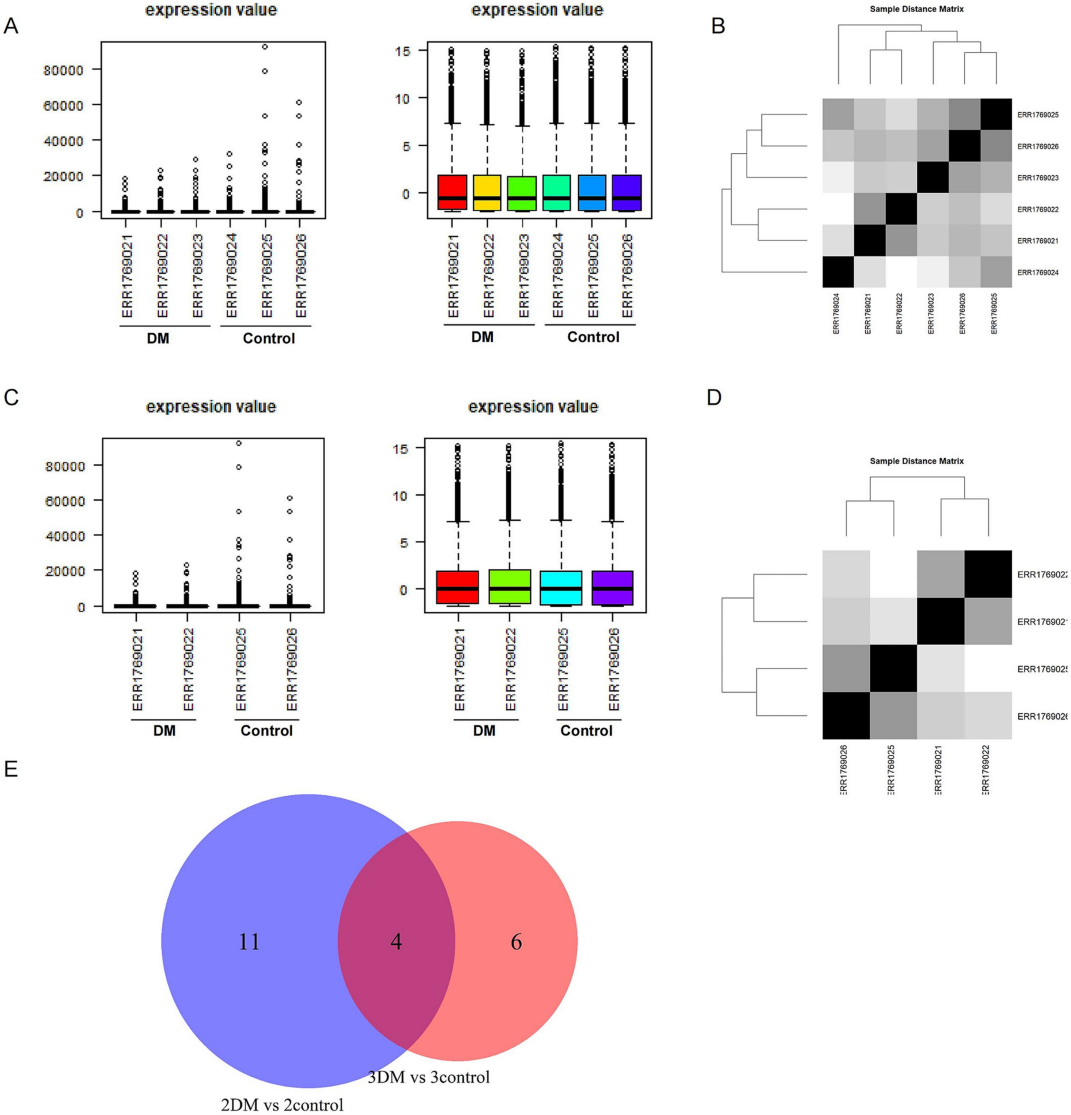
**(A)** Expression value of three DM patients and three control subjects before (left) and after (right) normalization; **(B)** Sample distance matrix of three DM patients and three control subjects; **(C)** Expression value of two DM patients and two control subjects before (left) and after (right) normalization; **(D)** Sample distance matrix of two DM patients and two control subjects; **(E)** Venn diagram of three DM patients vs. control subjects and two DM patients vs. 2 control subjects.

To obtain some candidate miRNAs for further experimental validation, we performed re-analysis of the data from PRJEB18466. First, based on the normalized data of three DM patients and three control subjects (Figures 1A,B), we found that the data ERR1769023 (control) and ERR1769024 (DM) were composed of the DM and control, respectively, suggesting that problems existed in patient selection. Therefore, we performed further analysis using the two DM patients and two control subjects (Figures 1C,D). We further minimized the candidate miRNAs by crossing the results of differentially expressed miRNAs from three DM patients vs. three control subjects and two DM patients vs. two control subjects. Finally, four miRNAs were selected for further experimental validation (Figure 1E), including miR-144-5p, miR-145-5p, miR-6805-3p, and miR-339-3p.

However, the results from expression quantification of these four miRNAs showed that miR-144-5p and miR-339-3p exhibited no difference among DKD, DM, and healthy controls (shown in Supplementary Figure S1). Therefore, miR-145-5p and miR-6805-3p were selected for further analysis.

**Figure 2:**
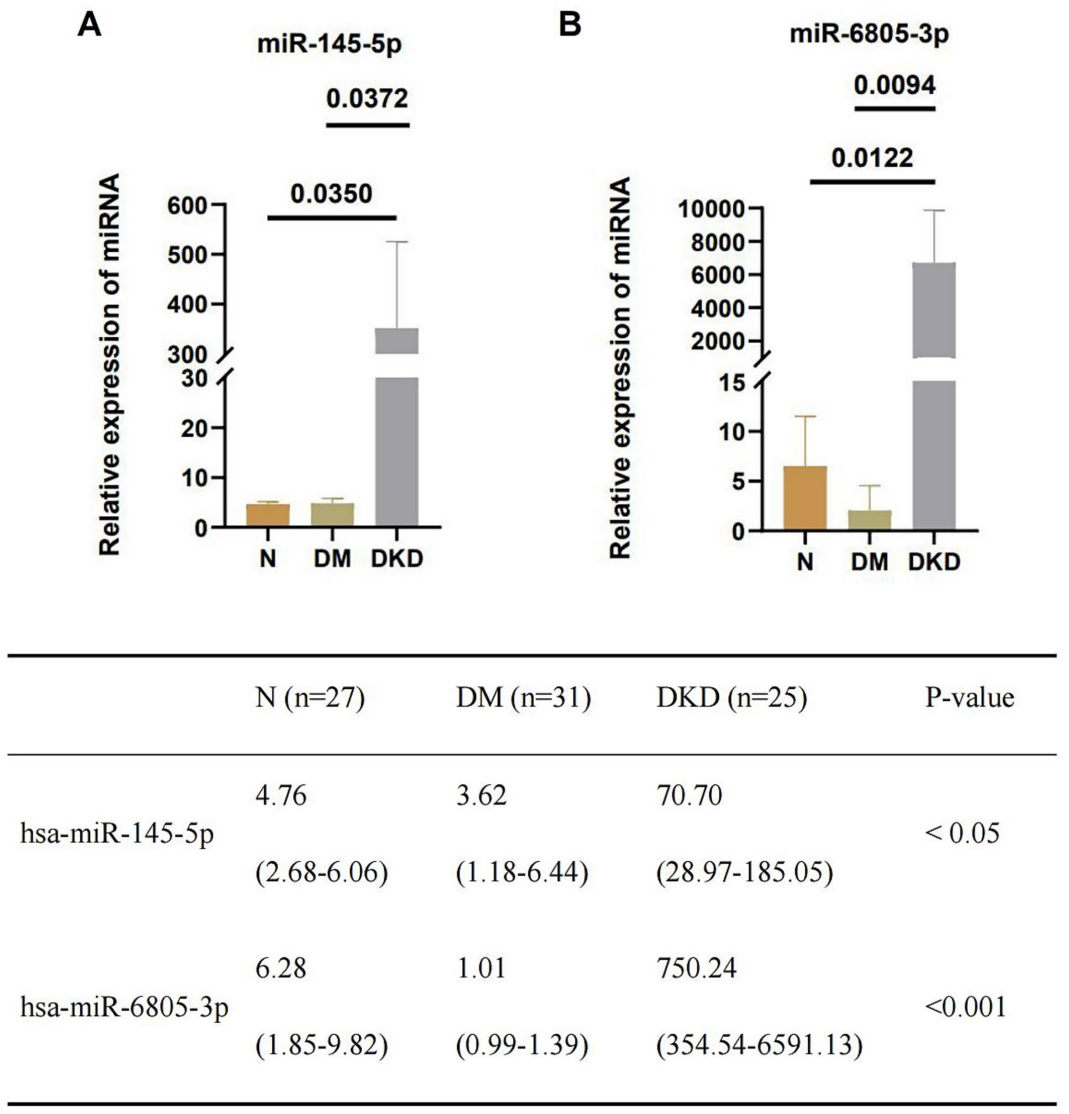
Upregulated level of platelet-derived **(A)** miR-145-5p and **(B)** miR-6805-3p from DKD compared to DM and healthy controls (N), respectively.

Upregulated Platelet-derived miR-145-5p and miR-6805-3p in DKD compared to DM and healthy controls. The expression levels of platelet-derived miR-145-5p and miR-6805-3p among the three groups were detected by RT-qPCR (shown in Figure 2), and the results showed that upregulated levels of miR-145-5p and miR-6805-3p were found in DKD compared to DM (*p* < 0.05) and the healthy control group (*p* < 0.001).

### 3.2 Correlation analysis between the levels of platelet-derived miR-145-5p and miR-6805-3p with clinical indices of DKD

**Figure 3:**
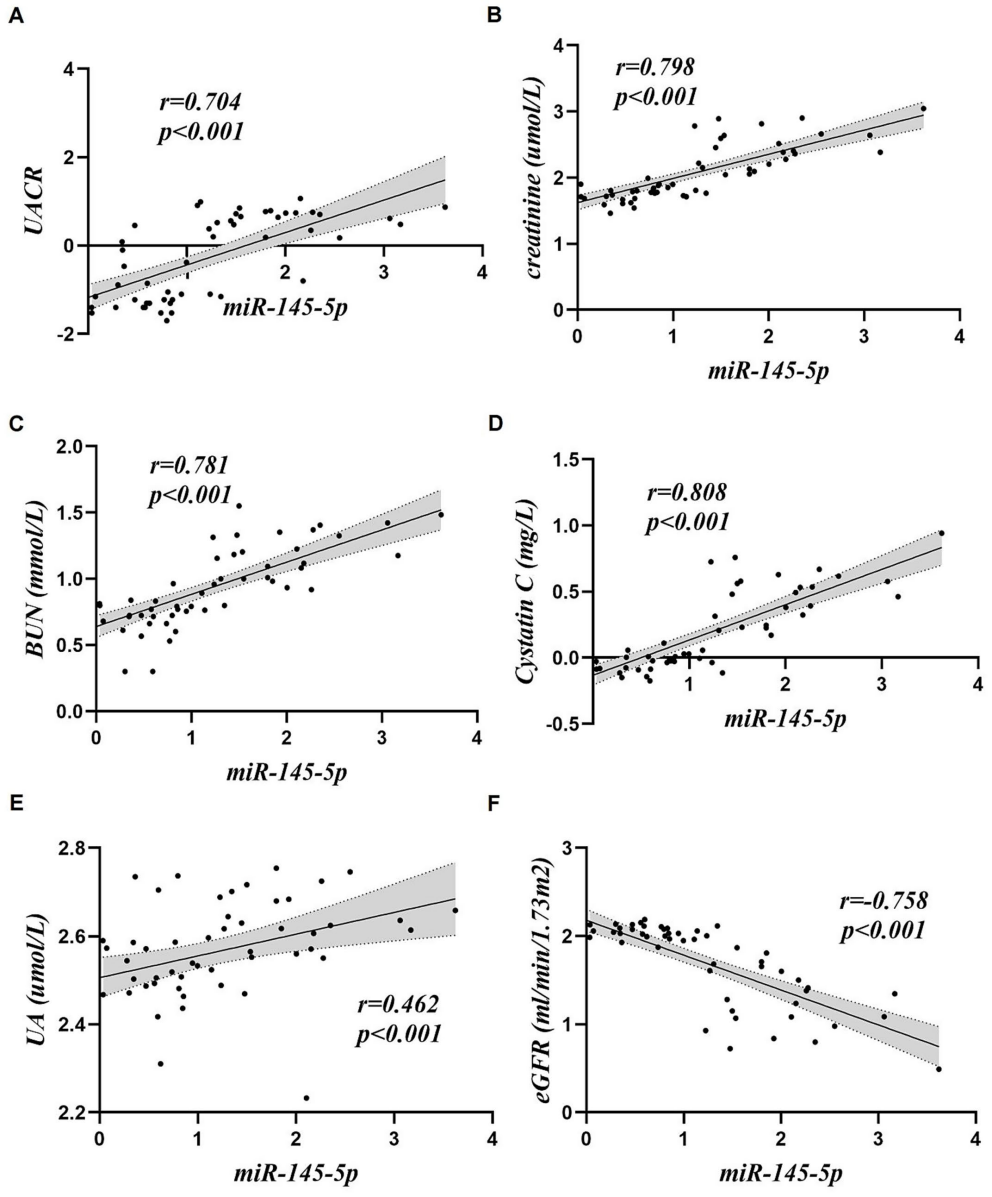
Correlation analysis of platelet-derived miR-145-5p with clinical indices. **(A)** UACR, **(B)** creatinine, **(C)** BUN, **(D)** Cystatin, **(E)** UA, **(F)** eGFR.

**Figure 4:**
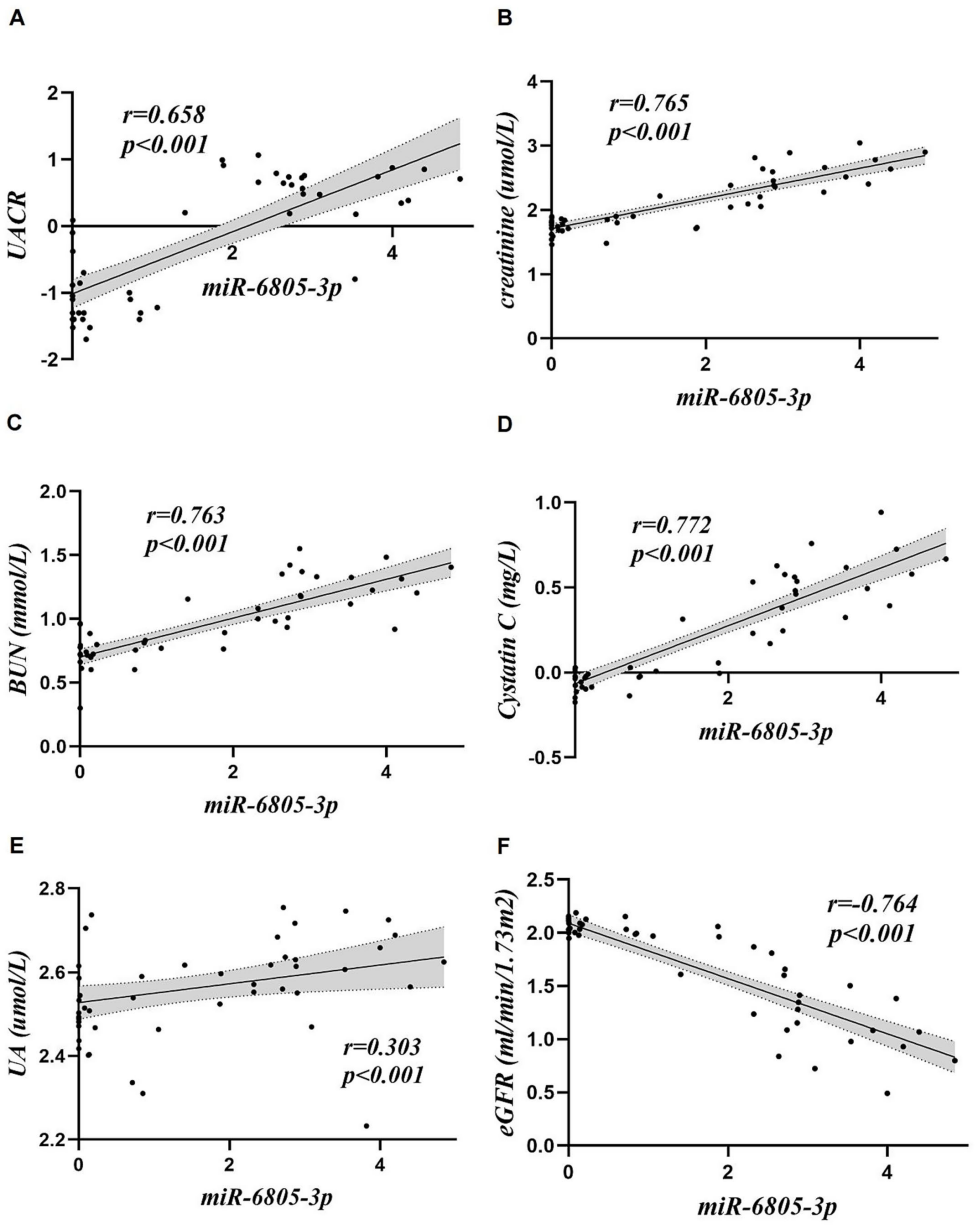
Correlation analysis of platelet-derived miR-6805-3p with clinical indices. **(A)** UACR, **(B)** creatinine, **(C)** BUN, **(D)** Cystatin, **(E)** UA, **(F)** eGFR.

Correlation analyses were performed using the expression levels of platelet-derived miR-145-5p and miR-6805-3p and the clinical indices of the included patients (shown in Table 2; Figures 3, 4). The results showed that platelet-derived miR-145-5p levels was positively correlated with UA, serum creatinine, BUN, UACR, cystatin C, globulin, PLR and NLR, and negatively correlated with eGFR, albumin, TC, LDL-C, blood glucose and HbA1c, with statistical significance (*p* < 0.05); in contrast, platelet-derived miR-6805-3p was positively correlated with UA, serum creatinine, BUN, UACR, cystatin C, PLR and NLR, and negatively correlated with eGFR, albumin, TC, LDL-C, blood glucose and HbA1c, with statistical significance (*p* < 0.05).

**Table 2:** Correlation analysis of miR-145-5p and miR-6805-3p expression with clinical indicators.

Clinical indicators	hsa-miR-145-5p		hsa-miR-6805-3p	
	r-value	*p*-value	r-value	*p*-value
Age (years)	0.154	0.256	0.226	0.100
TC (mmol/L)	−0.283	0.035*	−0.446	0.001*
TG (mmol/L)	0.026	0.851	−0.067	0.629
LDL-C (mmol/L)	−0.496	<0.001*	−0.565	<0.001*
HDL-C (mmol/L)	−0.209	0.121	−0.117	0.401
Blood glucose (mmol/L)	−0.401	0.002*	−0.441	0.001*
HbA1c (%)	−0.601	<0.001*	−0.583	<0.001*
UA (μmol/L)	0.462	<0.001*	0.303	0.026*
BUN (mmol/L)	0.781	<0.001*	0.763	<0.001*
Creatinine (μmol/L)	0.798	<0.001*	0.765	<0.001*
UACR	0.704	<0.001*	0.658	<0.001*
eGFR (CKD-EPI ml/min/1.73 m^2^)	−0.758	<0.001*	−0.764	<0.001*
Cystatin C (mg/L)	0.808	<0.001*	0.772	<0.001*
ALP (U/L)	−0.002	0.986	−0.013	0.927
Albumin (g/L)	−0.516	<0.001*	−0.568	<0.001*
Globulin (g/L)	0.296	0.027*	0.238	0.083
NLR	0.268	0.046*	0.437	0.001*
PLR	0.272	0.042*	0.352	0.009*
MHR	0.105	0.439	0.073	0.601
24-h urine protein (g/day)	−0.076	0.718	−0.155	0.481

*Indicates statistical significance, defined as a *p*-value < 0.05.

### 3.3 miR-6805-3p levels but not miR-145-5p differentiated DKD patients with different stages

Moreover, we also analyzed the expression levels of miR-145-5p and miR-6805-3p in different DKD stages, and the results are shown in Table 3. miR-6805-3p (*p* = 0.012) levels but not miR-145-5p (*p* = 0.118) had the ability for DKD staging differentiation. These data indicated the possible involvement of DM in the DKD progression of miR-6805-3p.

**Table 3:** miR-145-5p and miR-6805-3p levels in DKD patients with different stages.

Clinical indicator	Number of subjects (%)	Expression of mRNAs	
		miR-145-5p	miR-6805-3p
DKD stage (ml/min/1.73m^2^)			
G1 + G2 (>60)	4 (16%)	24.56 (13.07–61.86)	144.29 (75.30–318.71)
G3 (30–59)	6 (24%)	63.02 (19.86–113.35)	512.32 (145.51–2679.39)
G4 (15–29)	5 (20%)	181.34 (84.91–830.67)	767.81 (480.72–6827.82)
G5 (<15)	10 (40%)	105.93 (31.18–552.27)	5044.11 (690.55–18096.20)
*p*-value		0.118	0.012*

*Indicates statistical significance, defined as a *p*-value < 0.05.

## 4 Discussion

MicroRNAs (miRNAs) are a class of small non-coding RNAs that regulate gene expression by degrading or inhibiting the translation of their target messenger RNAs (14, 15). Since miRNA is a key regulator of cell homeostasis (16, 17), its dysfunction is an important part of cell and organ damage (18, 19). In DKD, miRNAs from different sources have been widely studied for their roles in the pathogenesis of DKD and serve as potential biomarkers for disease prediction (20). Peng et al. (21) found that urine-free miRNA-29a was significantly increased in DKD patients and was positively correlated with the degree of urinary albumin, which may be an alternative biomarker for DKD. Jia et al. (22) found that urinary exosome-derived miR-192 levels increased in the early DKD microalbuminuria group, suggesting that miR-192 may be a non-invasive tool for detecting early DKD in patients with T2DM. The results of a bioinformatic analysis showed that six miRNAs, including miR-21-5p, miR-29a-3p, etc., are involved in apoptosis, fibrosis, extracellular matrix accumulation, and other pathways associated with DKD pathogenesis and can be used as potential therapeutic targets against the progression of DKD (23). An animal study demonstrated that renal tissue-derived miRNA-337 increased expression in mice with DKD and caused podocyte damage by upregulating IL-6 and IL-18 levels (24). Obvious, recent advances in miRNA research have not only deepened understanding of the pathophysiological complexities of kidney disease, but also laid the foundation for the discovery of new diagnostic biomarkers (25), such as miR-3137, miR-4270 (26), miR-21, and miR-495 (27). However, until now, the miRNAs associated with DKD were basically derived from urinary exosomes, although several platelet-derived miRNAs have been shown to be early biomarkers of type 2 diabetes (T2DM) (28, 29), many platelet-derived miRNAs have been confirmed to be associated with diabetes (30–32), few studies have investigated whether platelet-derived miRNAs can be further used to evaluate the progression of DKD. Nührenberg et al. (33) performed miRNA analyses from the platelets of DM and controls, thereby providing the basis of our study.

miR-145-5p has been found to be associated with human cancers, regulating the proliferation of cancer cells, such as non-small cell lung cancer, colorectal adenocarcinoma, prostate cancer, and glioma (34–37). Urinary exosome-derived miR-145-5p was found to promote podocyte apoptosis by inhibiting Srgap2 and then activating RhoA/ROCK signaling pathway, suggesting that exosome miR-145-5p may play a role in the pathological process of DKD and serve as a non-invasive biomarker of DKD (38). Moreover, another study found that the target genes of miR-145-5p were located in the cytoskeleton and exosomes and were involved in 11 signaling pathways, including the mitogen-activated protein kinase (MAPK) pathway, transforming growth factor-beta (TGF-*β*) pathway, Forkhead Box O (FOXO) pathway, Ras pathway, and advanced glycation end-products (AGE)–receptor for AGE system (AGE–RAGE) pathway in diabetic complications (39). Among these pathways, TGF-β and MAPK signaling are considered to be the core of renal fibrosis in the late stage of DKD (40), which, in turn, suggests the possible role of miR-145-5p in renal fibrosis. Few studies have reported miR-6805-3p. Wang et al. showed that cancer cell-derived exosome contains miR-6805-3p, which had the ability to increase the expression of MAPK1, thereby promoting cervical cancer angiogenesis and tumor growth (41). A previous study (38) using urinary EVs also found that miR-145-5p was an upregulated pattern in DKD patients, which is consistent with our study. In this study, we found that platelet-derived miR-145-5p and miR-6805-3p were upregulated in DKD patients compared with DM and healthy control subjects, indicating a distinct DKD phenotype among DM patients. Moreover, we found that increased levels of platelet-derived miR-6805-3p were associated with DKD staging differentiation, suggesting its involvement in DM to DKD progression. miR-6805-3p was significantly higher than that in the N and DM groups, which was consistent with previous findings. There were some limitations in this study. First, due to time constraints, the sample size of the present study is relatively small, and all samples were from a single center. Second, the lack of long-term clinical follow-up and dynamic evaluation of miR-145-5p and miR-6805-3p levels fails to analyze the effects of miRNAs on patient prognosis. In addition, the limited availability of experimental tools, such as knock-out mice or kidney tissues, results in a failure to analyze the detailed mechanisms involved by these two miRNAs. In the near future, expanded clinical data with larger sample sizes and more in-depth mechanisms research are needed to verify the conclusions presented here and to explore the molecular mechanisms by which platelet-derived miRNAs affect the pathogenesis of DKD.

## 5 Conclusion

Platelet-derived miR-145-5p and miR-6805-3p may be novel non-invasive biomarkers for the diagnosis of DKD. Monitoring of miR-145-5p and miR-6805-3p may contribute to intervention and delay in the progression of DKD, based on their advantages, including simplicity, rapidity, and efficacy. In the near future, multicenter long-term longitudinal follow-up studies are needed to clarify their role in monitoring the condition and prognosis of DKD.

## Data Availability

The datasets presented in this study can be found in online repositories. The names of the repository/repositories and accession number(s) can be found in the article/Supplementary material.
