# Supplementary figures and images for: Expression and clinical significance of platelet-derived miR-145-5p and miR-6805-3p in diabetic kidney disease patients
Source: Front Med (Lausanne). 2026 Jan 12;12:1529759. doi: 10.3389/fmed.2025.1529759 (PMC12832840; doi:10.3389/fmed.2025.1529759)

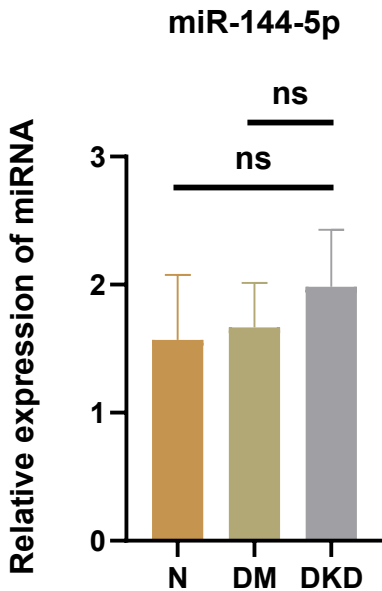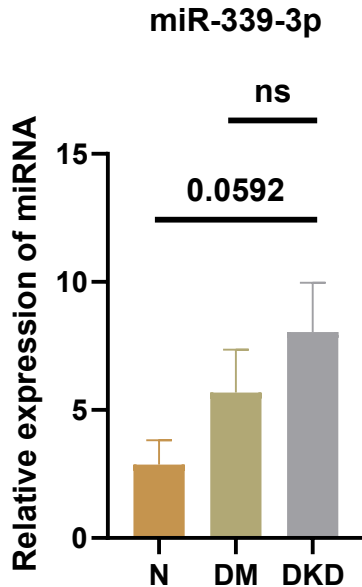

Supplement: Supplementary file 1 [file Data_Sheet_1.pdf]
