# Supplementary material for: Expression and clinical significance of platelet-derived miR-145-5p and miR-6805-3p in diabetic kidney disease patients
Source: Front Med (Lausanne). 2026 Jan 12;12:1529759. doi: 10.3389/fmed.2025.1529759 (PMC12832840; doi:10.3389/fmed.2025.1529759)
Supplement: Supplementary file 2 [file Data_Sheet_2.pdf]

	Forward	Reverse
miR-145-5p	5’-CGGTCCAGTTTTCCCAGGA-3’	5’-AGTGCAGGGTCCGAGGTATT-3’
miR-6805-3p	5’-TGCTCTGCTCCCCCGC-3’	5’-AGTGCAGGGTCCGAGGTATT-3’
miR-144-5p	5’-CGCGCGGGATATCATCATATAC-3’	5’-AGTGCAGGGTCCGAGGTATT-3’
miR-339-3p	5’-TGAGCGCCTCGACGACA-3’	5’-AGTGCAGGGTCCGAGGTATT-3’
U6	5’-GCTTCGGCAGCACATATACTAAAAT-3’	5’-CGCTTC ACGAATTTGCGTGTCAT-3’
RT-primer		
miR-144-5p	GTCGTATCCAGTGCAGGGTCCGAGGTATTCGCACTGGATACGACCTTACA	
miR-339-3p	GTCGTATCCAGTGCAGGGTCCGAGGTATTCGCACTGGATACGACCGGCTC	
